# Clinical manifestation and arthroscopic treatment of symptomatic posterior cruciate ligament cyst

**DOI:** 10.1186/s13018-018-0798-x

**Published:** 2018-04-13

**Authors:** Kai Tie, Hua Wang, Xinyu Zhao, Yang Tan, Jun Qin, Liaobin Chen

**Affiliations:** Department of Orthopaedic Surgery, Zhongnan Hospital of Wuhan University, 169# Donghu Rd, Wuchang District, Wuhan City, Hubei Provence People’s Republic of China

**Keywords:** Posterior cruciate ligament, Ganglion cyst, Symptom, Arthroscopic excision, Magnetic resonance imaging

## Abstract

**Background:**

Ganglion cyst of cruciate ligaments is a rare lesion; the prevalence is 0.3–0.8%. The purpose of this study was to present clinical features of symptomatic posterior cruciate ligament (PCL) cyst, introduce the arthroscopic excision technique, and evaluate the clinical outcome.

**Methods:**

A series of 11 patients with symptomatic PCL cyst from November 2012 to December 2014 were involved in this retrospective study. Detailed medical history collecting and physical examination were conducted. Magnetic resonance imaging (MRI) scan was used to confirm the diagnosis. Arthroscopic resection was performed, and the sample of the cyst was taken for pathologic examination. The follow-up averaged 30.7 months. International Knee Documentation Committee (IKDC) score, the range of motion (ROM), and MRI evaluations were obtained pre- and postoperatively to assess the surgical outcome. SPSS software was used for statistics analysis.

**Results:**

Eight males and 3 females with 6 left knees and 5 right knees were enrolled, the mean age was 34.4 years, and the duration of symptom was 19.0 months. All cases had a definite history of knee trauma or injury. The most common symptom was knee pain at flexion or in flexion-associated activities. MRI revealed the location and size of the cyst in each case. Pathologic examination showed the cyst wall was composed of dense fibroconnective tissue and widespread thick bundles of collagen, which is similar to the structure of ganglion cyst. At the final follow-up, MRI evaluation showed no cyst recurrence. The preoperative ROM and IKDC score were 2.3° to 108.6° and 40.5 ± 11.3, respectively, compared with the postoperative ROM and IKDC score which were 0° to 134.1° and 85.5 ± 4.8 (*p* < 0.05) separately.

**Conclusions:**

We conclude that the etiology of symptomatic PCL cyst is most likely associated with trauma, pain on flexion is a typical manifestation of symptomatic PCL cyst, MRI evaluation is an ideal examination for the diagnosis, and arthroscopic resection of symptomatic PCL cysts has a good outcome with no recurrence.

**Electronic supplementary material:**

The online version of this article (10.1186/s13018-018-0798-x) contains supplementary material, which is available to authorized users.

## Background

Ganglion cyst of cruciate ligaments is a rare lesion. The reported morbidity of cruciate ligament cyst is 0.36% by MRI examination and 0.8% by arthroscopy [1]. The lesion is commonly seen in people aged 20–40 years old and involves more males than females [1–3]. Compared with anterior cruciate ligament (ACL) cyst, posterior cruciate ligament (PCL) cyst is relatively seldom seen [3, 4]. Since not all PCL cysts are symptomatic, the reports of PCL cysts were hardly found until the wide utilization of MRI and arthroscopy two decades ago [5]. Most PCL cysts were incidentally detected by MRI or arthroscopy done for other knee lesions [2, 3, 6–8] in that symptomatic PCL cyst causing knee discomfort and restricted movements were exceedingly rare [4, 7].

Most scholars consider that the etiology of PCL cyst may be associated with trauma and chronic injury [6–9]. The main clinical manifestation includes knee pain, pain in extreme knee flexion, restricted movements, etc. [3]. Shetty et al. [7] have reported clinical manifestation of symptomatic PCL cysts as well as the process of arthroscopy-assisted operations. However, only a few studies have reported the surgical technique and outcomes of arthroscopic removal of PCL cyst. It is necessary to gain further insight into the entity of the disease, and the experience to diagnose and treat the lesion still needs accumulation. For this reason, we have retrospectively reviewed 11 cases of symptomatic PCL cyst treated with arthroscopy and systematically summarized the findings and experience in the aspects of clinical manifestation, MRI feature, arthroscopic operation, pathology, and the treatment outcome.

## Methods

All the patients with PCL cysts who underwent arthroscopy operation from November 2012 to December 2014 at Zhongnan Hospital of Wuhan University were retrospectively reviewed, and the patients who met the following inclusion and exclusion criteria in Table 1 were included. Eleven patients including 8 males and 3 females with the mean age of 34.4 years (ranging from17 to 53 years) were selected according to the inclusion criteria, six of whom had cysts in their left knees and five in their right knees (Additional file 1). The duration of the symptoms ranged from 3 to 96 months.

**Table 1:** Inclusion and exclusion criteria of patients

	Inclusion	Exclusion
Knee discomfort	✓	
Diagnosed as PCL cyst	✓	
Meniscus injury		✓
Ligament injury		✓
Cartilage injury		✓
Periarticular infection		✓

After medical history collecting, thorough physical examinations, routine X-ray, and MRI examination of the knee were performed. Also, preoperative knee function was evaluated using International Knee Documentation Committee (IKDC) score. The mean follow-up period was 30.7 months (range from19 to 53 months).

### Surgical techniques

**Fig. 1:**
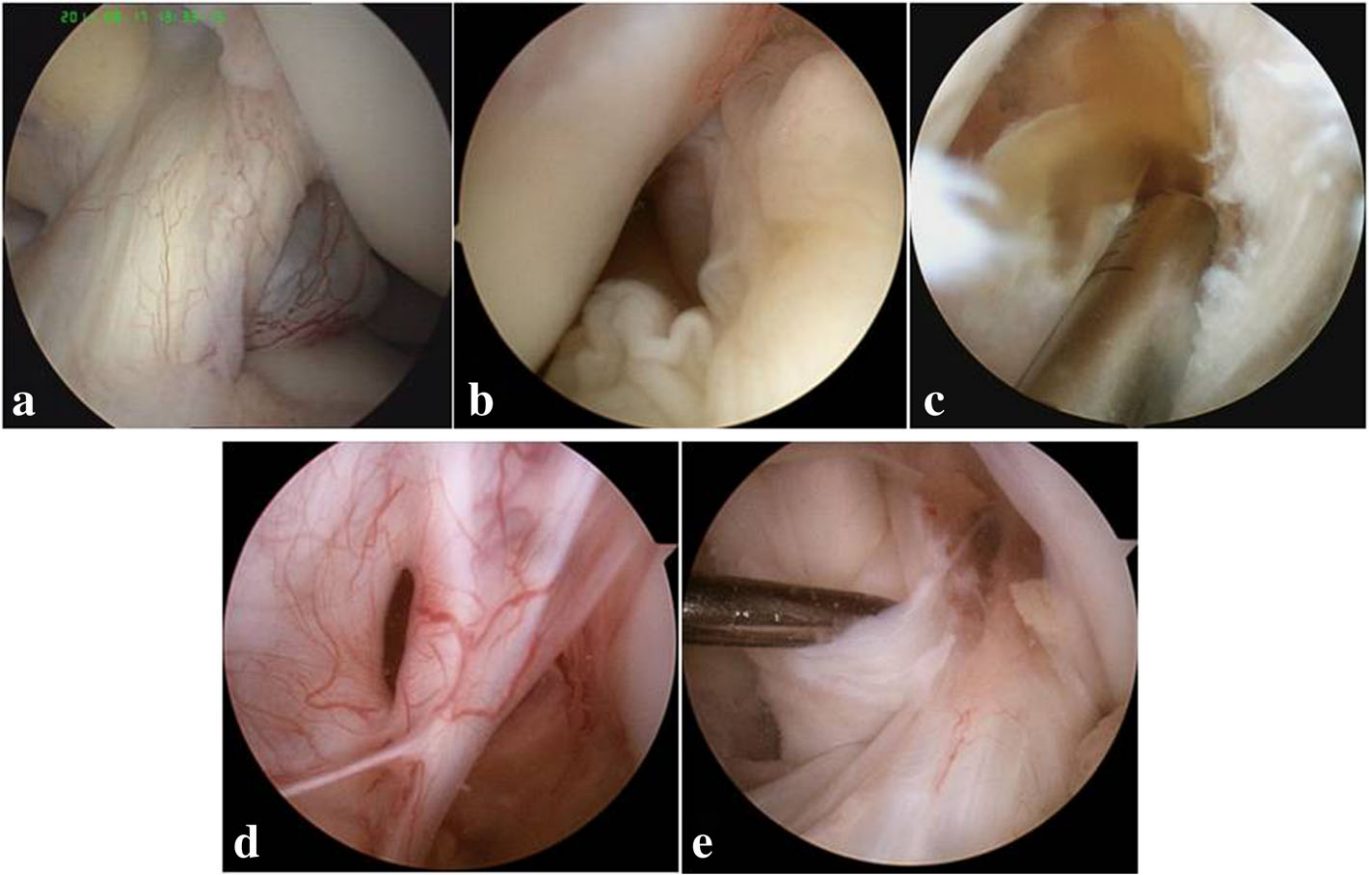
Arthroscopic image of the PCL cyst. **a** Viewing from the anterolateral portal, cyst which was partially behind the ACL encases the PCL and capillaries were distributed on the surface. **b** Viewing from the posteromedial portal, the PCL cyst was located posterior to the PCL and partly covered by synovium. **c** Viewing from the posteromedial portal, yellowish viscous fluid exuded out when the cyst was opened. **d** Capillaries were distributed on the inner surface of the cyst wall. **e** After resecting the front wall of the cyst, PCL was intact and uninvolved

Arthroscopy for every patient was performed by the same orthopedic surgeon by using the Linvatec arthroscope system. The patients were maintained in the supine position with epidural anesthesia, and a thigh tourniquet was used. A leg holder was to maintain the knee in the ROM of 90°. Routine arthroscopic examination of the knee joint was performed through standard anterolateral and anteromedial portals, while the posteromedial or posterolateral portal would also be established if it were necessary to visualize and manage the posterior compartment. Before excising the entire cyst, specimens were taken by using punch forceps for further pathologic examination. All the cysts were encased in elastic fibrous connective tissue without communicating with the joint cavity, and blood vessels were distributed on the surface of the cysts (Fig. 1a). Some of the cysts were covered by synovium tissue (Fig. 1b). Yellowish viscous fluid exuded out when the cysts were opened with surgical instruments (Fig. 1c). Capillaries were noted on the inner surface of the wall of the cysts (Fig. 1d). Most cysts were multilocular except two. In each patient, the specimen was taken for pathological examination and the whole cyst was resected by using the motorized shaver. Location distribution of the cysts was in accordance with the findings by MRI. Besides the four cysts which were found to encase the PCL, three cysts were located between the ACL and PCL, one of which was adjacent to the femoral insertion of the PCL, and the other four cysts were located posterior to the PCL, two of which were near the tibial insertion of the PCL. The PCL as well as ACL was intact (Fig. 1e), and the negative response was detected in each case by employing intraoperative drawer test.

### Rehabilitation protocol

Active circumduction of foot and ankle and straight leg raising were introduced immediately after surgery. During the fifth to seventh postoperative day, weight bearing was gradually increased from partial to full. The range of motion (ROM) was increased 30° per week for the next 3 weeks (total of 4 weeks reaching 90° flexion at the fourth week).

### Follow-up and evaluation

All the patients underwent clinical and radiographic assessments during the follow-up. Measurement of ROM and MRI scan were performed at the final follow-up after the operation. Moreover, the IKDC were used to evaluate the functional recovery.

### Statistical analysis

The statistical analysis was conducted with SPSS 21.0 for windows. Comparisons of IKDC score of knee functions between preoperative and postoperative were made with the paired-sample *t* test. All stated *p* values were two-tailed and considered significant when < 0.05.

## Results

### Clinical manifestation

Of the 11 patients, 7 complained of varying degrees of knee pain on active or passive flexion while 3 complained of knee pain on squatting and 1 had knee pain when climbing stairs. Five patients had definite history of knee trauma. Among them, one patient who experienced avulsion fracture of the tibial attachment of the PCL had been treated conservatively, and another one had MRI evidence of haematoma formation around PCL in the acute phase. Although the rests denied the obvious major traumatic event, they confirmed a history of chronic injuries when questioned in detail. For physical examination, hyperflexion pain was found in 10 cases while hyperextension pain also existed in 3 of them. Limited ROM was found in all the involved knees.

### Imaging feature

**Fig. 2:**
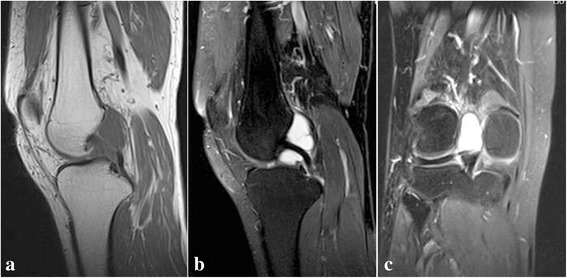
MRI images of the PCL cyst. **a** Sagittal T1W MRI: encasing the PCL with homogeneous low-signal intensity. **b** Sagittal T2W MRI: encasing the PCL with high-signal intensity. **c** Coronal T2W MRI: high-signal intensity

All patients including the one with a history of an avulsion fracture, presented normal images of knee joint through X-ray examination. The irregular or ovoid PCL cysts in the knee joints of the patients were shown on MRI image. All the cysts with recognizable membrane margin were well-defined with or without septum inside, presenting homogeneous low-signal intensity on T1W while high-signal intensity on both T2W and fat-suppressed T2-weighted SE. Being viewed in sagittal, coronal, and transverse plane, all the cysts were located either in the intercondylar notch encasing the PCL or in between ACL and PCL, or posterior to PCL (Fig. 2). Nine of the 11 patients presented multilocular cyst, and the other two patients were the unilocular cyst. The size of the cysts ranged from 10 × 8 × 6 mm to 51 × 30 × 21 mm.

### Pathologic examination

**Fig. 3:**
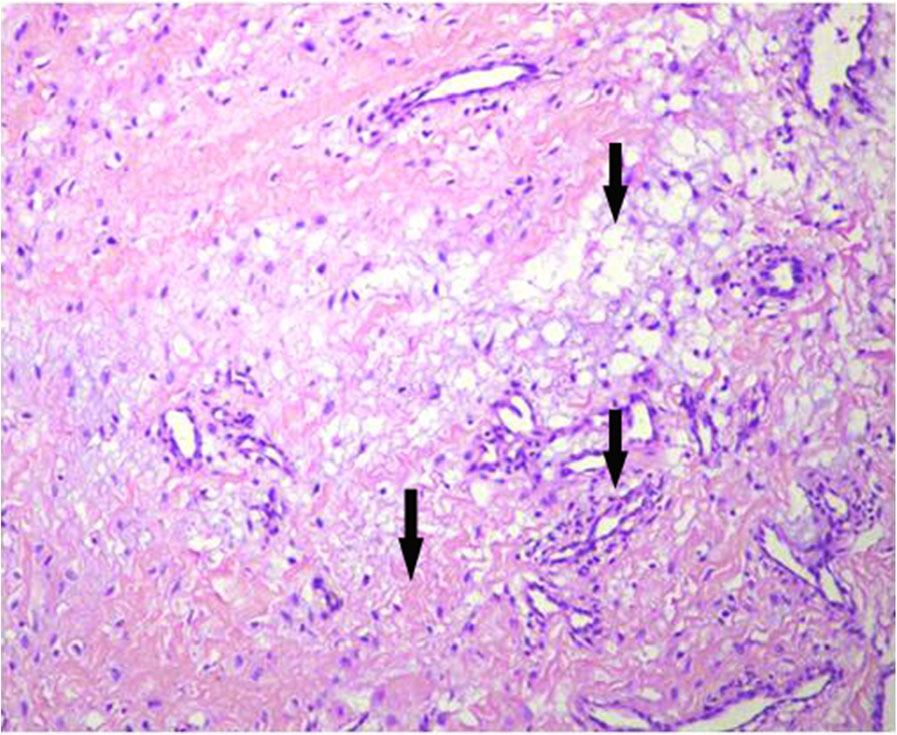
The HE staining of PCL cysts (× 200). Fibroplasia, massive capillary proliferation, and mucoid degeneration (indicated by black arrows) were observed in the cyst wall

HE staining showed that the cyst wall was composed of dense fibroconnective tissue and widespread thick bundles of collagen, the former of which containing stellate or spindle fibrocytes indicated inflammatory cell infiltration. In addition, fibroplasia and massive capillary proliferation were also observed in the cyst wall (Fig. 3). Three patients presented mucoid degeneration while another two showed lipoid degeneration.

### Clinical outcome

**Fig. 4:**
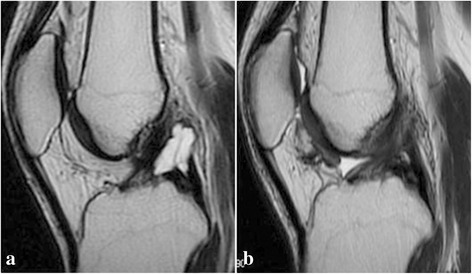
The preoperative and postoperative MRI images on a sagittal plane. **a** A multilocular PCL cyst. **b** There was no cyst recurrence at the final follow-up after operation

Postoperatively, all the patients obtained weight-bearing walking ability after 5 to 10 days and were asymptomatic after recovery. No sign of recurrence was found at the final follow-up after the operation by MRI examination (Fig. 4). At the final follow-up, the mean postoperative ROM and IKDC score were significantly increased than preoperative (Table 2).

**Table 2:** Pre- and postoperative ROM and IKDC score

	Preoperative	Postoperative
ROM(°)	106.4 ± 3.7	134.1 ± 1.8*
IKDC scores	40.5 ± 11.3	85.54.8*

*ROM* range of motion, *IKDC* International Knee Documentation Committee

*p < 0.05

## Discussion

Intra-articular ganglion cysts including PCL cyst are usually classified as “asymptomatic” and “symptomatic” [10, 11]. The cyst which is soley responsible for intermittent or chronic knee discomfort without any accompanied intra-articular lesions is defined as “symptomatic.” The cyst which has no clear etiology is difficult to be diagnosed clearly in time comparing with other intra-articular lesions or injuries in that it presents nonspecific clinical signs and symptoms. The results of this study showed that the most common manifestation of symptomatic PCL cyst was knee pain at flexion or in flexion-associated activities. Physical examination demonstrated that knee pain at hyperflexion and the resultantly reduced ROM appeared in almost all the patients. Literatures showed that the changes in the length and torsion of PCL induced by knee flexion might cause traction or compression on cysts, which may stimulate the nerve endings on synovium and result in limited ROM secondary to pain and abnormal sensation [1, 2, 12, 13]. Previous studies reported that the clinical manifestations of symptomatic PCL cyst might include arthralgia, joint swelling and effusion, and restricted ROM induced by pain [1–4, 6–8, 12, 14–17]. Our results are similar to the above reports. And furthermore, the findings of this series of cases indicate that knee pain at flexion may be the specific clinical manifestation of symptomatic PCL cyst.

Since MRI examination could sensitively and specifically identify the anatomic and morphologic interrelation of the synovial tissues with their surrounding structures without invasion, it has been suggested as the primary choice for diagnosing cystic lesions of the knees [18–20]. The cyst showed by MRI exhibits well-defined and homogeneous low-signal intensity on T1W, high-signal intensity both on T2W, and fat-suppressed T2-weighted SE, while the cystic wall manifests middle or low signal in T2W [1–3, 7, 8, 12, 17, 21–23]. The PCL cysts are usually single or multiloculated ones who are separated by soft tissue [8]. The MRI pictures in our series of cases were in accordance with these imaging features. The precise location of the PCL cyst is important for planning an arthroscopic approach. At present, posteromedial or posterolateral portals are not routine approach and the posterior compartment is not visualized regularly. Once the cyst is located posterior to PCL, it would be difficult to found the cyst for subsequent diagnosis. Therefore, the preoperative MRI scan is essential.

Although the pathogenesis of ganglion cysts of cruciate ligament is still unknown, most literature showed that trauma and chronic injuries played a key role in cyst formation [2, 3, 6, 7]. Bui-Mansfield et al. [18] found that 50% of the cruciate ganglion cysts had intra-articular lesion and degeneration, indicating that the cysts were associated with trauma and slight chronic injuries. Shetty et al. [7] reported that most cysts contained yellowish viscous fluid, while only a few cysts had bloody liquid. Lunhao et al. [6] explained that the cysts with bloody viscous liquid were associated with trauma, which resulted in a lesion of the PCL, synovial hernia, and formation of the hematoma. Along with the course of extension, the synovial tissue encases the hematoma and form a cyst gradually. In the early period, the content is bloody liquid and the hematoma would be absorbed and organized. The size of cyst increases as the synovial tissue keeps secreting mucus. At this stage, the contents turned to yellowish viscous fluid due to the breakdown of hemoglobin, while the cysts with transparent liquid contents coincided with a chronic injury. In the current study, 5 of the 11 patients had a definite history of trauma. The others memorized their experience of antecedent knee trauma or repeated slight knee injuries when questioned in detail. The findings in our study provided further evidence to support that the etiology of PCL cysts is closely associated with trauma and chronic injury. Pathologic examination of HE staining revealed inflammatory cell infiltration, widespread thick bundles of collagen, fibroplasia, and massive capillary proliferation in cyst wall. The inner surface of the cyst was covered by synoviocytes. Mucoid and lipoid degeneration, ruptured bundles of collagen (Fig. 3), were also found. All these indicated that the formation of PCL cyst might be associated with trauma which subsequently induced a synovial hernia and the degeneration of ligament secondary to trauma and chronic injury.

Treatment of ganglion cysts of cruciate ligaments mainly includes arthroscopic resection, ultrasound-, and CT-guided joint paracentesis [6, 7, 24]. However, postoperative recurrence occurred more often after paracentesis alone than after a complete resection [24]. The reason is that the cystic wall will persist and it may recur as the aspirating hole acts as a one-way valve and the synovium continues secreting mucus after paracentesis. While arthroscopy could excise the cyst completely and manage other lesions at the same time, the recurrence is rare after a complete excision [7, 9, 16, 24]. In the present study, all cases were performed the arthroscopic excision of the PCL cysts, and no recurrence was observed on postoperative MRI images at final follow-up. The double posteromedial approach for the arthroscopic resection of PCL cyst in this study, which was different from the posterior trans-septal portal [9], had a good clinical outcome.

The present study is the first to systematically describe the findings of the symptoms, imaging diagnosis, etiology, pathologic changes, and arthroscopic treatments of PCL cyst. There were still several limitations of our study, such as the small number of patients, the retrospective design, and the lack of comparative control group, which need to be further improved in future research.

## Conclusions

In conclusion, the etiology of symptomatic PCL cyst may be associated with trauma and chronic injuries. The knee pain at flexion could be considered as a typical manifestation of symptomatic PCL cyst. MRI examination is an ideal method for diagnosing PCL cyst in time. And arthroscopic resection of PCL cysts would lead to a good outcome for patients without any recurrence.

## Additional file

Additional file 1: **Table S1.** Clinical characteristics of the 11 patients. (DOCX 17 kb)

## Abbreviations

IKDC: International Knee Documentation Committee
PCL: Posterior cruciate ligament
ROM: Range of motion
