# Supplementary material for: Clinical manifestation and arthroscopic treatment of symptomatic posterior cruciate ligament cyst
Source: J Orthop Surg Res. 2018 Apr 13;13:84. doi: 10.1186/s13018-018-0798-x (PMC5899343; doi:10.1186/s13018-018-0798-x)
Supplement: Supplementary file 1 — Table S1. Clinical characteristics of the 11 patients. (DOCX 17 kb) [file 13018_2018_798_MOESM1_ESM.docx]

**Table S1** Clinical characteristics of the 11 patients

|  | Gender | Age  (year) | Duration of symptoms  (months) | History | Symptom | Physical examination | Location of cyst | Size (mm) | Preoperative ROM(°) | Preoperative IKDC score | Follow-up time  （month） | Postoperative ROM(°) | Postoperative IKDC score |
| --- | --- | --- | --- | --- | --- | --- | --- | --- | --- | --- | --- | --- | --- |
| 1 | M | 28 | 96 | Trauma | Pain when going upstairs and downstairs，  Powerless knee | Positive McMurray test, medial joint line tenderness | Between ACL and PCL | 10×8×6；13×8×8 | 5-90 | 16 | 39 | 0-130 | 81 |
| 2 | M | 53 | 12 | Chronic injures | Restricted flexion due to pain，Dull pain when walking | Restricated knee motion due to pain, Hyperflexion and hyperextension pain | Surrounding PCL | 34×20×18 | 0-100 | 21 | 49 | 0-135 | 82 |
| 3 | M | 43 | 6 | Chronic injures | Pain on squatting | Hyperflexion pain | Surrounding PCL | 36×16×13 | 3-115 | 48 | 53 | 0-140 | 89 |
| 4 | M | 33 | 24 | Chronic injures | Pain on squatting | Hyperflexion and hyperextension pain | Between ACL and PCL | 25×16×14 | 0-115 | 45 | 20 | 0-125 | 82 |
| 5 | M | 24 | 4 | Trauma (Old avulsion fracture of the tibial attachment of the PCL) | Pain on flexion, limitation of flexion due to pain | Hyperflexion and hyperextension pain | Posterior to PCL | 20×14×13 | 0-110 | 47 | 36 | 0-140 | 93 |
| 6 | F | 45 | 12 | Chronic injures | Pain on flexion, limitation of flexion due to pain | Hyperflexion pain | Surrounding PCL | 36×22×14 | 7-105 | 38 | 10 | 0-135 | 85 |
| 7 | M | 17 | 3 | Sports injures | Pain on flexion | Hyperflexion pain | Posterior to PCL near PCL’s tibial insertion | 20×13×14 | 0-110 | 44 | 34 | 0-135 | 83 |
| 8 | M | 42 | 12 | Chronic injures | Pain on flexion | Hyperflexion pain | Posterior to PCL | 31×20×10 | 10-105 | 44 | 19 | 0-125 | 79 |
| 9 | F | 22 | 6 | Chronic injures | Pain on flexion and squatting | Hyperflexion pain | Surrounding PCL | 51×30×21 | 0-115 | 47 | 22 | 0-140 | 85 |
| 10 | M | 35 | 24 | Trauma | Pain on flexion and squatting,  Pain when going upstairs and downstairs | Hyperflexion pain | Between ACL and PCL | 27×22×21 | 0-100 | 48 | 23 | 0-130 | 93 |
| 11 | F | 36 | 10 | Trauma | Pain when going downstairs and squatting | Hyperflexion pain | Posterior to PCL near PCL’s tibial insertion | 15×10×8 | 0-130 | 47 | 33 | 0-140 | 89 |
